# RhoA/ROCK Signaling Pathway Mediates Shuanghuanglian Injection-Induced Pseudo-allergic Reactions

**DOI:** 10.3389/fphar.2018.00087

**Published:** 2018-02-12

**Authors:** Jiayin Han, Yong Zhao, Yushi Zhang, Chunying Li, Yan Yi, Chen Pan, Jingzhuo Tian, Yifei Yang, Hongyu Cui, Lianmei Wang, Suyan Liu, Jing Liu, Nuo Deng, Aihua Liang

**Affiliations:** Institute of Chinese Materia Medica, China Academy of Chinese Medical Sciences, Beijing, China

**Keywords:** Shuanghuanglian injection, pseudo-allergic reactions, RhoA/ROCK signaling pathway, vascular leakage, endothelial permeability

## Abstract

**Background:** Shuanghuanglian injection (SHLI) is a famous Chinese medicine used as an intravenous preparation for the treatment of acute respiratory tract infections. In the recent years, the immediate hypersensitivity reactions induced by SHLI have attracted broad attention. However, the mechanism involved in these reactions has not yet been elucidated. The present study aims to explore the characteristics of the immediate hypersensitivity reactions induced by SHLI and deciphers the role of the RhoA/ROCK signaling pathway in these reactions.

**Methods:** SHLI-immunized mice or naive mice were intravenously injected (i.v.) with SHLI (600 mg/kg) once, and vascular leakage in the ears was evaluated. Passive cutaneous anaphylaxis test was conducted using sera collected from SHLI-immunized mice. Naive mice were administered (i.v.) with a single dose of 150, 300, or 600 mg/kg of SHLI, and vascular leakage, histamine release, and histopathological alterations in the ears, lungs, and intestines were tested. *In vitro*, human umbilical vein endothelial cell (HUVEC) monolayer was incubated with SHLI (0.05, 0.1, or 0.15 mg/mL), and the changes in endothelial permeability and cytoskeleton were observed. Western blot analysis was performed and ROCK inhibitor was employed to investigate the contribution of the RhoA/ROCK signaling pathway in SHLI-induced hypersensitivity reactions, both in HUVECs and in mice.

**Results:** Our results indicate that SHLI was able to cause immediate dose-dependent vascular leakage, edema, and exudates in the ears, lungs, and intestines, and histamine release in mice. These were pseudo-allergic reactions, as SHLI-specific IgE was not elicited during sensitization. In addition, SHLI induced reorganization of actin cytoskeleton and disrupted the endothelial barrier. The administration of SHLI directly activated the RhoA/ROCK signaling pathway both in HUVECs and in the ears, lungs, and intestines of mice. Fasudil hydrochloride, a ROCK inhibitor, ameliorated the SHLI-induced hypersensitivity reactions in both endothelial cells and mice indicating its protective effect. SHLI-induced pseudo-allergic reactions were mediated by the activation of the RhoA/ROCK signaling pathway.

**Conclusion**: This study presents a novel mechanism of SHLI-induced immediate hypersensitivity reactions and suggests a potential therapeutic strategy to prevent the associated adverse reactions.

## Introduction

Shuanghuanglian injection (SHLI) is a famous antiviral and antimicrobial Chinese medicine. The intravenous preparation of SHLI is derived from the extracts of three herbal plants, namely, *Flos lonicerae japonicae, Radix scutellariae*, and *Fructus forsythia* (Yan et al., 2010; Liu et al., 2011). As a therapeutic drug, SHLI shows great curative effects with low drug resistance. Hence, it is widely used in the treatment of acute respiratory tract infections, including laryngitis, pharyngitis, tonsillitis, rhinitis, bronchiolitis, and pneumonia (Zhang et al., 2013; Gao et al., 2014; Fang et al., 2015). However, adverse drug reactions (ADRs) are reported in approximate 3.25% of patients treated with SHLI (Wang et al., 2010). Retrospective analysis of electronic databases reveal that 70% of ADRs associated with SHLI treatment are due to hypersensitivity reactions, which affect a number of organs and tissues including the skin, gastrointestinal tract, and respiratory system, along with systemic anaphylaxis and anaphylactic shock (Li, 2010; Shang, 2014).

Pseudo-allergic reactions are non-immune-mediated hypersensitivity reactions that represent one type of unpredictable ADRs (Waller, 2011). These reactions are usually indistinguishable from an IgE-mediated allergic reaction, but they lack immunological specificity (Warrington and Silviu-Dan, 2011; Demoly et al., 2014). In these reactions, non-immune mechanisms trigger effector cells such as mast cells and basophils to release histamine and other inflammatory mediators (McNeil et al., 2015; Pichler and Hausmann, 2016). The common clinical symptoms of pseudo-allergic reactions, also present in IgE-mediated allergic reactions include urticaria, angioedema, bronchospasm, gastrointestinal signs, and anaphylaxis (Jurakic Toncic et al., 2009; Spoerl et al., 2017). However, unlike allergic reactions, pseudo-allergic reactions initiate at the first exposure to stimulus without prior sensitization (Wang H. et al., 2011; Farnam et al., 2012). Results based on studies conducted by various medical centers indicate that most SHLI-mediated hypersensitivity reactions occur in patients on the first exposure, while in over 50% of cases, these reactions occur within 1 h following drug administration (Wu and Zhang, 2007; Li, 2010; Yang et al., 2014). Hence, most SHLI hypersensitivity reactions are postulated to be pseudo-allergic reactions.

RhoA and its downstream effector Rho-associated kinase (ROCK) regulate the formation of stress fibers (Hall, 1998; Jaffe and Hall, 2005; Pellegrin and Mellor, 2007) and influence endothelial permeability (van Nieuw Amerongen et al., 2007). RhoA acts as a molecular switch cycling between an active GTP-bound state and an inactive GDP-bound state (Etienne-Manneville and Hall, 2002). Binding of GTP-RhoA activates ROCK, which directly phosphorylates myosin light chain (MLC) and suppresses the activity of MLC phosphatase. This results in enhanced expression of phosphorylated myosin light chain (p-MLC), which triggers the assembly of stress fibers, increases actomyosin contractility, and causes endothelial barrier dysfunction (Riento and Ridley, 2003; van Nieuw Amerongen et al., 2007; Mong and Wang, 2009; Tojkander et al., 2012). The RhoA/ROCK signaling pathway plays an important role in endothelial hyperpermeability induced by a variety of stimulus including thrombin (van Nieuw Amerongen et al., 2000), vascular endothelial growth factor (Hoang et al., 2004), tumor necrosis factor-α (Peng et al., 2011), high glucose (Zhao et al., 2015), histamine (Mikelis et al., 2015), mustard oil (Chen et al., 2011), and penicillin (Han et al., 2016). Major symptoms associated with SHLI hypersensitivity reactions including rash and/or pruritus, facial edema, nausea and/or vomiting, abdominalgia, diarrhea, chest distress and/or dyspnea, asthma, cough, and anaphylactic shock (Wang et al., 2010) would likely be consequences secondary to increased vascular leakage. In the present study, we investigated the role of the RhoA/ROCK signaling pathway in SHLI-mediated hypersensitivity reactions using both *in vivo* mouse model and *in vitro* experiments. In addition, we studied the effect of ROCK specific inhibitor on SHLI-induced vascular hyperpermeability. Our results might provide new insights for the prevention and treatment of SHLI-induced hypersensitivity reactions.

## Materials and Methods

### Ethics Statement

This study was carried out in accordance with the recommendations of ethical guidelines and regulations for the use of laboratory animals and cells issued by the Institute of Chinese Materia Medica, China Academy of Chinese Medical Sciences, Beijing, China. All animal-related procedures adhered to the protocol approved by the Institutional Animal Care and Use Committee of the Institute of Chinese Materia Medica, China Academy of Chinese Medical Sciences.

### Reagents

Shuanghuanglian injection was obtained from a hospital. Aluminum hydroxide gel (AHG) was purchased from Thermo Fisher Scientific. Ovalbumin (OVA), FITC-dextran (MW 4 × 10^4^), and rhodamine-phalloidin were obtained from Sigma–Aldrich. Fasudil hydrochloride was acquired from Chase Sun Pharmaceutical. Rabbit anti-p-MLC2 (Thr18/Ser19) (3674), rabbit anti-MLC2 (3672), rabbit anti-p-MYPT1 (Thr 696) (5163), rabbit anti-MYPT1 (2634), and rabbit anti-RhoA (67B9) (2117) were purchased from Cell Signaling Technology. Activated RhoA pull down assay kit was obtained from Cytoskeleton. Histamine assay kit was purchased from Immuno-Biological Laboratories.

### Animals

Male ICR mice were purchased from Vital River Laboratory Animal Technology, Co., Ltd. (Beijing, China). Mice were at 8–10 weeks of age and randomly divided into experimental groups on a body weight-stratified basis. Animals were kept under specific-pathogen-free conditions.

### Cell Culture

Human umbilical vein endothelial cells (HUVECs) were kindly provided by Dr. Song (Academy of Military Sciences, Beijing, China), who purchased them from American Type Culture Collection (ATCC, Manassas, VA, United States). Cells were used between passages 10–15 and were cultured in Dulbecco’s modified Eagle’s medium (DMEM) supplemented with 10% fetal bovine serum (FBS) at 37 °C in a 5% CO_2_ incubator (HERAcell, Heraeus, Hanau, Germany).

### Sensitization Test

The recommended clinical dose of SHLI is 60 mg/kg. In the present study, mice were given a dose of 600 mg/kg, calculated as per the equation for the conversion of animal doses (FDA, 2005).

During the sensitization phase, mice received intraperitoneal injection (i.p.) of a mixture containing equal volume of SHLI and AHG (SHLI/AHG) (300 mg/kg), normal saline/AHG (NS/AHG) or OVA (20 mg/kg) on days 1, 3, and 5.

Vascular leakage was assessed on day 19. Briefly, immunized mice were challenged by intravenous slow injection (i.v.) (approximately 2 min) of a mixture containing equal volume of SHLI and Evans blue (EB) (SHLI/EB) at 600 mg/kg or normal saline/EB (NS/EB). The naive mice received a single dose (i.v.) of SHLI/EB (600 mg/kg) or NS/EB. Thirty minutes after drug/EB administration, SHLI-induced changes on vascular permeability were analyzed by measuring the plasma protein leakage. EB extravasation in each ear was given a score of 1 to 6 based on the blue staining area (Han et al., 2016). The ears of each mouse were then preserved in formamide for EB extraction. The amount of EB extravasation was determined at an absorption wavelength of 610 nm using a microplate reader (Thermo Scientific Varioskan Flash, Thermo Scientific, United States).

Passive cutaneous anaphylaxis (PCA) test was performed on day 19. Immunization sera were collected and 50 μL of serially diluted sera (1, 1/2, 1/4, and 1/8) was intradermally injected into the dorsal skin of recipient mice. Two hours later, the recipient mice were challenged by administration (i.v.) of SHLI/EB (600 mg/kg), NS/EB, or OVA/EB (40 mg/kg). After 30 min, the mice were observed for EB extravasation into the subcutaneous tissue. When the blue spot in the dorsal region had a diameter of 5 mm or more, the PCA test result was considered positive.

### Evaluation of Vascular Leakage and Histological Examination

Naive mice were treated (i.v.) with a single dose of NS/EB or SHLI/EB at 150, 300, or 600 mg/kg. Vascular leakage was assessed as described in the previous section. Punch biopsies of the left ears (8 mm diameter) were weighted and complete left ears were preserved in formamide for EB extraction. The right ears, lungs, and intestines were preserved in 10% neutral-buffered formalin, embedded in paraffin, sectioned, and stained with hematoxylin and eosin (H&E). Slides were viewed using an Olympus BX53 microscope (Olympus, Tokyo, Japan) and images were obtained by Cell Sens Standard v 1.9 digital imaging software (Olympus).

In order to confirm the role of the RhoA/ROCK signaling pathway in SHLI-induced vascular leakage, mice were injected (i.p.) with 30 mg/kg fasudil hydrochloride once daily for three consecutive days. Thirty minutes after the last dose of fasudil, 600 mg/kg SHLI/EB was administrated (i.v.). In parallel groups, mice were treated (i.p.) with normal saline for 3 days and then administered (i.v.) 600 mg/kg SHLI/EB or NS/EB. Assessment of vascular leakage and histopathological evaluation were performed as described above.

### Histamine Detection

Mice were injected (i.v.) with normal saline or 600 mg/kg SHLI. Five minutes after drug administration, blood samples were withdrawn and centrifuged at 3000 × *g* at 4°C for 15 min. Histamine concentration in the plasma was analyzed using the mouse histamine assay kit according to the manufacturer’s instructions.

### Assessment of Endothelial Permeability Using Transwell Assay

Endothelial permeability was assessed using FITC-dextran flux measurements. HUVECs were grown in a transwell insert chamber (0.4 μm, Millicell, Merck Millipore, Cork, Ireland) until a confluent endothelial monolayer was formed. The cells were stimulated with SHLI (0.05, 0.1, or 0.15 mg/mL) for 1 h. Another group of cells were pretreated with 10 μM fasudil hydrochloride for 30 min and then incubated with 0.15 mg/mL SHLI for 1 h. The concentrations of SHLI used for stimulation corresponded to its equivalent concentration in the blood. These *in vitro* experimental concentrations of SHLI were derived from values converted from the *in vivo* blood concentrations where its major components were detected (Zeng et al., 2010; Zhou et al., 2013). In addition, the values were lower than the 5% maximal inhibitory concentration (IC_5_). For the assessment of endothelial monolayer permeability, 400 μL DMEM containing FITC-dextran (1 mg/mL) was loaded in the upper part of the chamber. FITC-dextran that diffused through the cells was collected from the lower part of the chamber after 10, 20, 30, 40, 50, and 60 min, respectively. The fluorescence in the lower chamber was determined using a microplate reader (Thermo Scientific Varioskan Flash). Fluorescent apparent permeability coefficient (P_app_, cm/s) of the samples was calculated (Wang L.Y. et al., 2011).

### Fluorescence Staining

Rhodamine-phalloidin staining was used for visualizing F-actin. Endothelial monolayers or HUVECs were cultured on a 48-well plate and incubated with SHLI (0.05, 0.1, or 0.15 mg/mL) for 1 h, or pretreated with 10 μM fasudil hydrochloride for 30 min and then stimulated with 0.15 mg/mL SHLI for 1 h. Cells were then stained with 5 μg/mL rhodamine-phalloidin for 1 h at 37°C. Cells were visualized and images were obtained using Olympus IX71 fluorescent microscope (Olympus) and DP2-BSW v 2.2 software (Olympus).

### Preparation of Tissue and Cell Lysates

*In vivo*, animals were administered (i.v.) 600 mg/kg SHLI, or pretreated with 30 mg/kg fasudil hydrochloride (i.p.) and then injected (i.v.) with SHLI as described above. Ears, lungs, and intestines were collected from single SHLI-treated animals at 15, 30, or 60 min after SHLI administration and from fasudil-pretreated mice at 30 min after SHLI injection. Tissues were homogenized in lysis buffer and lysates were centrifuged at 12,000 × *g* at 4°C for 10 min. Supernatants were stored at -80°C for western blot assay.

*In vitro*, HUVECs were incubated with 0.15 mg/mL SHLI for 15, 30, or 60 min, or pretreated with 10 μM fasudil hydrochloride for 30 min and then stimulated with 0.15 mg/mL SHLI for 30 min. Cells were collected and lysed at the scheduled time points. The supernatants of lysates were acquired as mentioned above.

Activity of RhoA was assessed by a pull down assay according to the manufacturer’s instructions. Briefly, GTP-RhoA was purified from the supernatants of lysates from the ears, lungs, and intestines of mice or HUVECs with the Rho binding domain region of rhotekin protein bound to glutathione-sepharose beads. The GTP-RhoA thus obtained was analyzed by western blot assay.

### Immunoblot Analysis

Equal amounts of protein (50 μg) were loaded and separated on sodium dodecyl sulfate polyacrylamide gel (SDS-PAGE) and transferred onto polyvinylidene difluoride (PVDF) membranes. The membranes were incubated with respective primary antibodies of anti-p-MLC2 (1:500), anti-MLC2 (1:500), anti-p-MYPT1 (1:500), anti-MYPT1 (1:500), and anti-RhoA (1:500) at 4°C over-night. After washing the membranes, they were incubated with the corresponding secondary antibodies at room temperature for 2 h. Protein bands were visualized using enhanced chemiluminescence detection substrate.

### Statistical Analysis

Data are expressed as mean (M) ± standard error of mean (SEM). Quantitative data were analyzed using the one-way analysis of variance (ANOVA) method. The score of vascular leakage of the ear was analyzed using the Rank-test. Statistical analyses were performed with SPSS 16.0 software. *p* < 0.05 was considered statistically significant.

## Results

### SHLI Induces IgE-Independent Vascular Leakage

**FIGURE 1:**
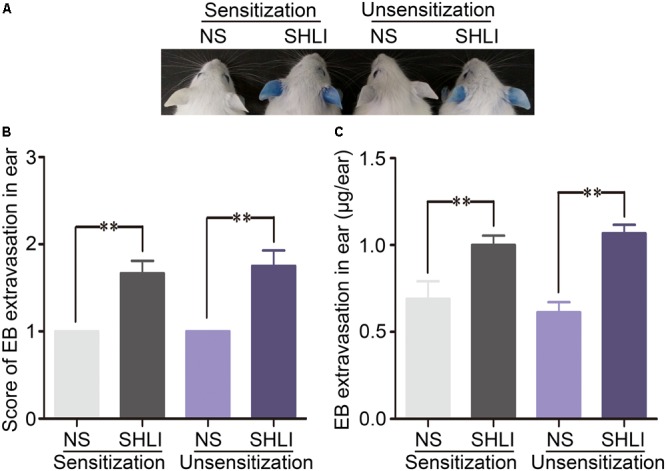
Vascular leakage in Shuanghuanglian injection (SHLI)-immunized and naive mice administered with a single intravenous injection (i.v.) of SHLI/Evans blue (EB). **(A)** Vascular leakage in SHLI-immunized mice challenged with (i.v.) SHLI/EB once and naive mice administered with a single injection of SHLI/EB. **(B)** Score of EB extravasation in the ears (*n* = 6 per group). **(C)** The amount of EB extravasation in the ears (*n* = 6 per group). Data are expressed as M ± SEM; ^∗^*p* < 0.05, ^∗∗^*p* < 0.01 compared with the normal saline-treated group.

Evans blue binds plasma albumin forming a firm albumin-EB complex and is widely employed as a marker to detect vascular leakage. In the present study, the extent of vascular permeability was evaluated by assessing EB extravasation in the ears of mice. In the vehicle control groups, none of the mice exhibited visible blue staining, indicating the inability of EB to induce vascular leakage on its own. However, significantly enhanced EB extravasation was observed in the ears of animals treated (i.v.) with SHLI/EB. This reaction was initiated approximately 10–15 min after administration (i.v.) of SHLI/EB and peaked by 25 min. Interestingly, EB extravasation was similar in SHLI-immunized and unimmunized mice (**Figures 1A–C**). Prior sensitization with SHLI had no effect on this vascular hyperpermeability reaction. These results suggest the role of non-immune mechanisms in eliciting vascular leakage caused by SHLI.

In order to confirm the absence of the involvement of immune mechanisms in SHLI-induced vascular leakage, IgE-mediated PCA test was performed. Significant blue spots were observed in all OVA-treated animals (the positive control group). However, mice administered with SHLI showed no dermal response (**Table 1**) indicating that SHLI could not elicit IgE production in mice. These results confirm that SHLI-induced vascular leakage was a non-immune-mediated pseudo-allergic reaction.

**Table 1:** Results of PCA reaction after the injection of drug/Evans blue (EB).

Test article	Sensitized dose (mg/kg)	Challenge dose (mg/kg)	Positive reaction (*n* = 6, %)
Normal saline	0	0	0
OVA	20	40	100
SHLI	300	600	0

**FIGURE 2:**
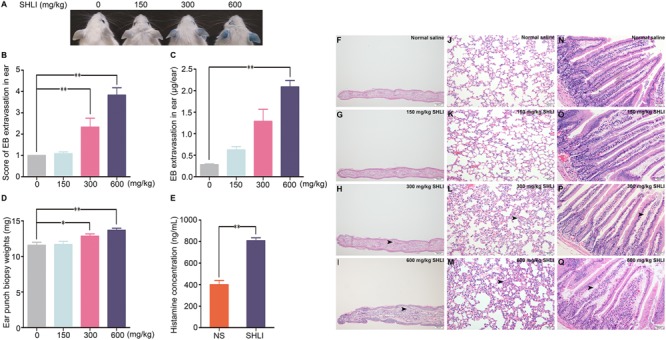
Dose-dependent vascular leakage and pathological changes, and histamine release in naive mice received a single intravenous injection (i.v.) of Shuanghuanglian injection (SHLI)/Evans blue (EB). **(A)** Dose-dependent vascular leakage in naive mice received a single injection of SHLI/EB at different dose levels. **(B)** Score of EB extravasation in the ears (*n* = 6 per group). **(C)** The amount of EB extravasation in the ears (*n* = 6 per group). **(D)** Weights of the ear punch biopsies (*n* = 6 per group). **(E)** Histamine concentration in the plasma (*n* = 6 per group). **(F)** Microscopic examination of ears. It showed no obvious gross histopathological change (normal saline). **(G)** Microscopic examination of ears. It showed no obvious gross histopathological change (150 mg/kg SHLI). **(H)** Microscopic examination of ears. Slight thickening, edema, and neutrophil infiltration (arrow head) were observed (300 mg/kg SHLI). **(I)** Microscopic examination of ears. Moderate thickening, edema, and neutrophil infiltration (arrow head) were observed (600 mg/kg SHLI). **(J)** Microscopic examination of lungs. It showed no obvious gross histopathological change (normal saline). **(K)** Microscopic examination of lungs. It showed no obvious gross histopathological change (150 mg/kg SHLI). **(L)** Microscopic examination of lungs. Slight interstitial edema and alveolar wall widening (arrow head) were observed (300 mg/kg SHLI). **(M)** Microscopic examination of lungs. Mild interstitial edema and alveolar wall widening (arrow head) were observed (600 mg/kg SHLI). **(N)** Microscopic examination of intestines. It showed no obvious gross histopathological change (normal saline). **(O)** Microscopic examination of intestines. It showed no obvious gross histopathological change (150 mg/kg SHLI). **(P)** Microscopic examination of intestines. Mild edema and exudates (arrow head) were observed (300 mg/kg SHLI). **(Q)** Microscopic examination of intestines. Moderate edema and exudates (arrow head) were observed (600 mg/kg SHLI). Data are expressed as M ± SEM; ^∗^*p* < 0.05, ^∗∗^*p* < 0.01 compared with the normal saline-treated group.

To understand better the role of SHLI-induced pseudo-allergic reactions, dose-dependent effect of SHLI/EB was analyzed *in vivo* in mice. Thirty minutes after a single injection of SHLI/EB, significant dose-dependent vascular hyperpermeability was observed (**Figure 2A**). The staining area and the amount of EB extravasation closely correlated with the dose of SHLI/EB administered (**Figures 2B,C**). SHLI also caused an increase in the ear weight of mice, which was about 11.2 and 18.1% in mice administered with 300 and 600 mg/kg SHLI, respectively, compared to the normal saline group (**Figure 2D**). In addition, SHLI triggered augmented histamine release in mice (**Figure 2E**), a feature generally seen in clinical pseudo-allergic reactions (Ring et al., 1999, 2014; McNeil et al., 2015).

As SHLI-induced ADRs are typically seen in the skin, gastrointestinal tract, and respiratory system, we examined the pathological alterations in the ears, lungs, and intestines of SHLI-treated mice. Consistent with increased vascular leakage, apparent dose-dependent ear thickening, edema, and neutrophil infiltration were observed in the ears (**Figures 2F–I**). In a dose-dependent manner, SHLI induced the swelling of pneumocytes and looseness of cytoplasm resulting in interstitial edema and alveolar wall-widening (**Figures 2J–M**). Microscopic examination of intestines revealed exudates and edema (**Figures 2N–Q**).

### SHLI Induces Endothelial Hyperpermeability and Activates RhoA/ROCK Signaling Pathway in HUVECs

**FIGURE 3:**
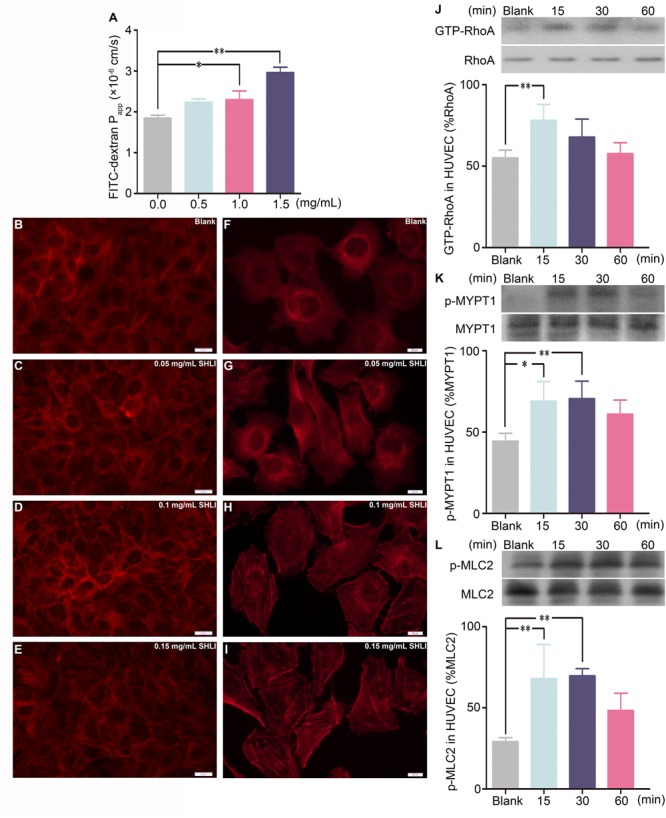
Shuanghuanglian injection (SHLI)-induced alterations in endothelial monolayer permeability, F-actin distribution, and expression of GTP-RhoA, p-MYPT1, and p-MLC2 in a time-dependent manner in human umbilical vein endothelial cells (HUVECs). **(A)** Fluorescence apparent permeability coefficient of FITC-dextran through the HUVEC monolayer (*n* = 3, per group). F-actin formation and distribution in HUVEC monolayer (**B**: untreated cells; **C**: 0.05 mg/mL SHLI; **D**: 0.1 mg/mL SHLI; **E**: 0.15 mg/mL SHLI). F-actin formation and distribution in HUVECs (**F**: untreated cells; **G**: 0.05 mg/mL SHLI; **H**: 0.1 mg/mL SHLI; **I**: 0.15 mg/mL SHLI). **(J)** Protein level of GTP-RhoA in HUVECs incubated with 0.15 mg/mL SHLI (*n* = 3, per group). **(K)** Protein level of p-MYPT1 in HUVECs incubated with 0.15 mg/mL SHLI (*n* = 3, per group). **(L)** Protein level of p-MLC2 in HUVECs incubated with 0.15 mg/mL SHLI (*n* = 3, per group). Blank: untreated cells. Data are expressed as M ± SEM; ^∗^*p* < 0.05, ^∗∗^*p* < 0.01 compared with untreated cells.

Vascular permeability is closely related to endothelial barrier function. Hence, we examined the effect of SHLI on HUVECs. The role of SHLI in endothelial permeability was analyzed by Transwell assay. In addition, cells were stained with rhodamine-phalloidin to visualize F-actin. Stimulation with SHLI for 1 h significantly enhanced the diffusion of FITC-dextran through a confluent endothelial monolayer in a concentration-dependent manner. The permeability coefficient of FITC-dextran was significantly increased in SHLI-treated groups than in the control group (**Figure 3A**). Consistent with the endothelial monolayer permeability assay, SHLI also evoked reorganization of actin cytoskeleton. Compared to the basal state represented by few thin stress fibers occasionally present at the cell periphery, stimulation with SHLI induced reorganization and assembly of F-actin to form thicker stress fibers that were diffused across the cells (**Figures 3B–I**).

Endothelial barrier dysfunction accompanied by formation and distribution of stress fibers is associated with the activation of RhoA (van Nieuw Amerongen et al., 1998, 2000). In order to study the role of the RhoA/ROCK signaling pathway in SHLI-induced endothelial barrier disruption, the protein levels of GTP-RhoA, p-MYPT1, and p-MLC2 in HUVECs stimulated with SHLI were analyzed. SHLI activated the RhoA/ROCK signaling pathway in a time-dependent manner. The expression of GTP-RhoA was up-regulated within 15 min and decreased by 60 min of SHLI stimulation (**Figure 3J**). Correspondingly, expression of p-MYPT1 and p-MLC2 were significantly enhanced by 15 min and peaked at 15–30 min after SHLI treatment (**Figures 3K,L**).

### Fasudil Attenuates SHLI-Induced Alterations in Endothelial Monolayer

**FIGURE 4:**
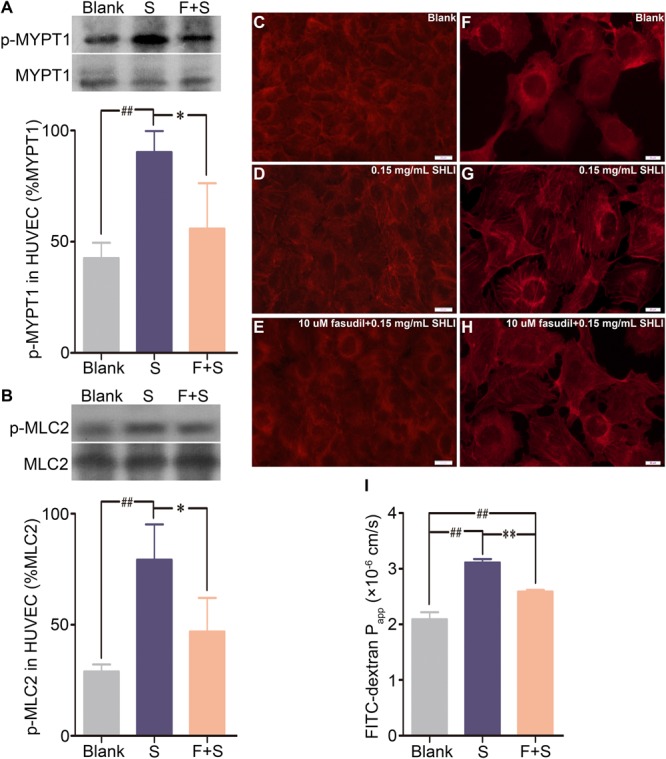
Inhibitory effect of fasudil hydrochloride on Shuanghuanglian injection (SHLI)-induced up-regulation of p-MYPT1 and p-MLC2, F-actin reorganization, and endothelial monolayer hyperpermeability in HUVECs. **(A)** Protein level of p-MYPT1 in HUVECs (*n* = 3, per group). **(B)** Protein level of p-MLC2 in HUVECs (*n* = 3, per group). F-actin formation and distribution in HUVEC monolayer (**C**: untreated cells; **D**: 0.15 mg/mL SHLI; **E**: 10 μM fasudil hydrochloride + 0.15 mg/mL SHLI). F-actin formation and distribution in HUVECs (**F**: untreated cells; **G**: 0.15 mg/mL SHLI; **H**: 10 μM fasudil hydrochloride + 0.15 mg/mL SHLI). **(I)** Fluorescence apparent permeability coefficient of FITC-dextran through the HUVEC monolayer (*n* = 3, per group). Blank: untreated cells; S: 0.15 mg/mL SHLI; F + S: 10 μM fasudil hydrochloride + 0.15 mg/mL SHLI. Data are expressed as M ± SEM; ^∗^*p* < 0.05, ^∗∗^*p* < 0.01 compared with 0.15 mg/mL SHLI-treated cells; ^#^*p* < 0.05, ^##^*p* < 0.01 compared with untreated cells.

In order to confirm the involvement of the RhoA/ROCK signaling pathway in SHLI-induced endothelial changes, we additionally addressed the effect of fasudil, a specific inhibitor of ROCK, on SHLI-mediated breakdown of endothelial function. Preincubation of HUVECs with fasudil hydrochloride significantly decreased the over expression of p-MYPT1 and p-MLC2 elicited by SHLI (**Figures 4A,B**). In addition, SHLI-induced rearrangement of actin cytoskeleton and increased formation and distribution of stress fibers were inhibited by fasudil (**Figures 4C–H**). Fasudil hydrochloride significantly decreased the enhanced permeability coefficient induced by SHLI, thereby manifesting a protective effect as was evident in the fasudil pretreatment group compared to the single SHLI-treated group (**Figure 4I**).

### The RhoA/ROCK Signaling Pathway Mediates SHLI-Induced Pseudo-allergic Reactions

**FIGURE 5:**
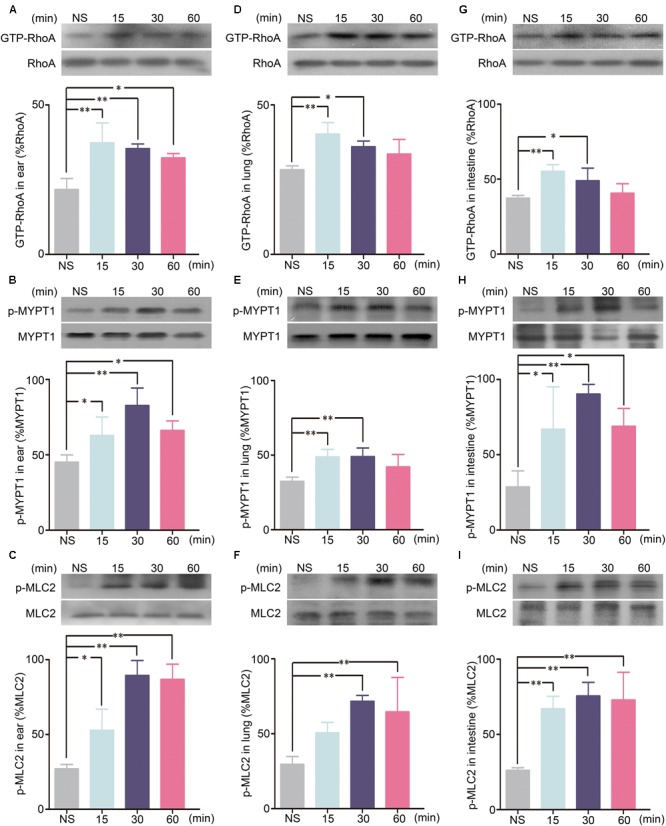
GTP-RhoA, p-MYPT1, and p-MLC2 expressions for different time courses in the ears, lungs, or intestines of mice received a single intravenous injection (i.v.) of 600 mg/kg Shuanghuanglian injection (SHLI). **(A)** Protein level of GTP-RhoA in the ears of mice (*n* = 3, per group). **(B)** Protein level of p-MYPT1 in the ears of mice (*n* = 3, per group). **(C)** Protein level of p-MLC2 in the ears of mice (*n* = 3, per group). **(D)** Protein level of GTP-RhoA in the lungs of mice (*n* = 3, per group). **(E)** Protein level of p-MYPT1 in the lungs of mice (*n* = 3, per group). **(F)** Protein level of p-MLC2 in the lungs of mice (*n* = 3, per group). **(G)** Protein level of GTP-RhoA in the intestines of mice (*n* = 3, per group). **(H)** Protein level of p-MYPT1 in the intestines of mice (*n* = 3, per group). **(I)** Protein level of p-MLC2 in the intestines of mice (*n* = 3, per group). Data are expressed as M ± SEM; ^∗^*p* < 0.05, ^∗∗^*p* < 0.01 compared with the normal saline-treated group.

To determine the role of the RhoA/ROCK signaling pathway in SHLI-induced pseudo-allergic reactions, the protein levels of GTP-RhoA, p-MYPT1, and p-MLC2 in the ears, lungs, and intestines of mice administered with SHLI were analyzed. Our results suggested activation of the RhoA/ROCK signaling pathway by SHLI as the expression of GTP-RhoA, p-MYPT1, and p-MLC2 in the ears, lungs, and intestines were significantly elevated after 15 min of SHLI administration and mostly remained consistently high for 15–60 min post-administration (**Figures 5A–I**). The time course of these augmented proteins correlated with the manifestation of vascular leakage in mice treated with SHLI, indicating a possible relation between the two processes.

**FIGURE 6:**
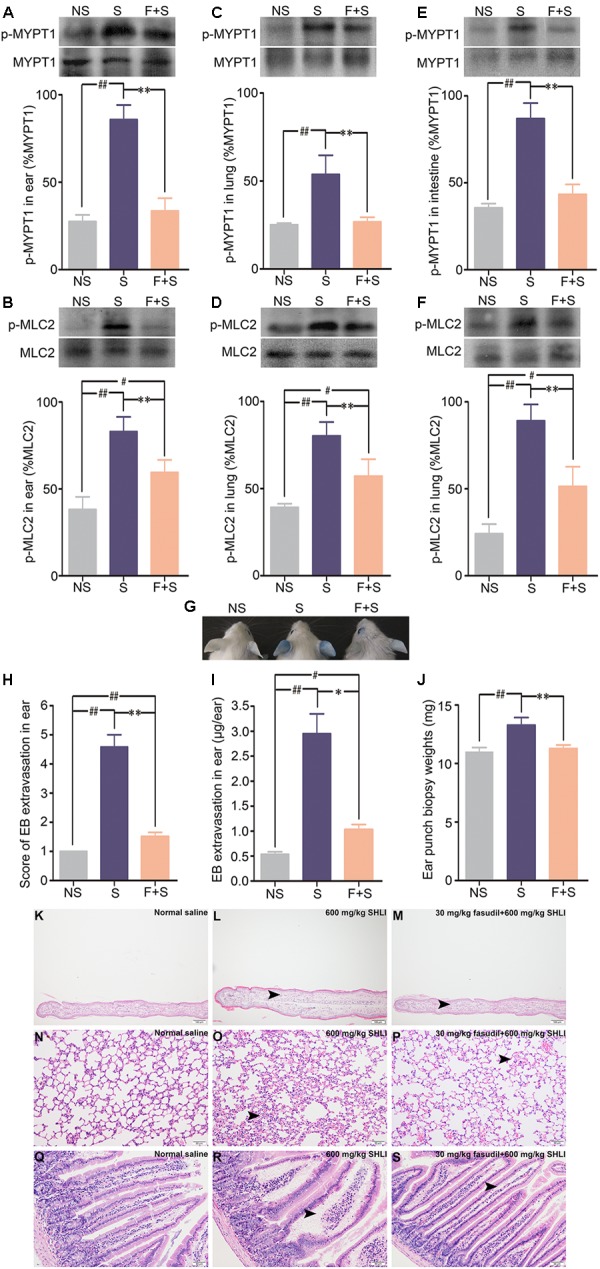
Inhibitory effect of fasudil hydrochloride on Shuanghuanglian injection (SHLI)-induced up-regulation of p-MYPT1 and p-MLC2 in the ears, lungs, and intestines of mice and pseudo-allergic reactions in mice. **(A,B)** Protein levels of p-MYPT1 and p-MLC2 in the ears of mice (*n* = 3, per group). **(C,D)** Protein levels of*(p-MYPT1 and p-MLC2 in the lungs of mice (*n* = 3, per group). **(E,F)** Protein levels of p-MYPT1 and p-MLC2 in the intestines of mice (*n* = 3, per group). **(G)** Vascular leakage in mice received a single intravenous injection of 600 mg/kg SHLI/Evans blue (EB), or pretreated with 30 mg/kg fasudil hydrochloride and injected with 600 mg/kg SHLI/EB. **(H)** Score of EB extravasation in the ears (*n* = 6 per group). **(I)** The amount of EB extravasation in the ears (*n* = 6 per group). **(J)** Weights of the ear punch biopsies (*n* = 6 per group). **(K)** Microscopic examination of ears. It showed no obvious gross histopathological change (normal saline). **(L)** Microscopic examination of ears. Moderate thickening, edema, and neutrophil infiltration (arrow head) were observed (600 mg/kg SHLI). **(M)** Microscopic examination of ears. Slight thickening, edema, and neutrophil infiltration (arrow head) were observed (30 mg/kg fasudil hydrochloride + 600 mg/kg SHLI). **(N)** Microscopic examination of lungs. It showed no obvious gross histopathological change (normal saline). **(O)** Microscopic examination of lungs. Mild interstitial edema and alveolar wall widening (arrow head) were observed (600 mg/kg SHLI). **(P)** Microscopic examination of lungs. Slight interstitial edema and alveolar wall widening (arrow head) were observed (30 mg/kg fasudil hydrochloride + 600 mg/kg SHLI). **(Q)** Microscopic examination of intestines. It showed no obvious gross histopathological change (normal saline). **(R)** Microscopic examination of intestines. Moderate edema and exudates (arrow head) were observed (600 mg/kg SHLI). **(S)** Microscopic examination of intestines. Slight edema and exudates (arrow head) were observed (30 mg/kg fasudil hydrochloride + 600 mg/kg SHLI). S: 600 mg/kg SHLI. F + S: 30 mg/kg fasudil hydrochloride + 600 mg/kg SHLI. Data are expressed as M ± SEM; ^∗^*p* < 0.05, ^∗∗^*p* < 0.01 compared with 600 mg/kg SHLI-treated group; ^#^*p* < 0.05, ^##^*p* < 0.01 compared with normal saline-treated group.)*

To confirm the above findings, the effect of ROCK inhibitor in abrogating SHLI-induced pseudo-allergic reactions was analyzed. Protein assay results indicated that fasudil hydrochloride inhibited the enhanced expression of p-MYPT1 and p-MLC2 in the ears, lungs, and intestines of mice induced by SHLI administration (**Figures 6A–F**). Accordingly, mice treated with fasudil prior to administration of SHLI were obviously resistant to vascular dysfunction evoked by SHLI (**Figure 6G**). In addition, enhanced EB extravasation seen in the ears of mice and increase in the ear weight caused by SHLI were significantly alleviated by fasudil hydrochloride (**Figures 6H–J**). The histopathological examination also confirmed that SHLI-induced edema and exudates in the ears, lungs, and intestines were improved by the inhibitor. Remission of pathological alterations in the ear, alveolar wall, and intestinal villa were evidently observed in the fasudil pretreatment group compared to the single SHLI-treated group (**Figures 6K–S**).

## Discussion

The results of *in vivo* and *in vitro* experiments in the present study indicate the ability of SHLI to induce pseudo-allergic reactions by activating the RhoA/ROCK signaling pathway. SHLI is a commonly prescribed drug in Chinese hospitals and clinics for the treatment of acute upper respiratory tract infections. However, the use of SHLI is associated with the risk of ADRs. From 2011 to 2016, the ADRs of SHLI, particularly the most commonly observed hypersensitivity reactions were specifically listed in the *China State Food and Drug Administration Annual Adverse Reactions Report for Drugs*. The symptoms associated with SHLI hypersensitivity reactions mainly involve the skin, gastrointestinal, and respiratory disorders (Wang et al., 2010), all of which are caused by increased vascular leakage. The ADRs of SHLI limit its therapeutic use. The underlying mechanism of ADRs induced by SHLI has not yet been elucidated. Limited researches have indicated the ability of SHLI to induce hypersensitivity reactions in rats (Li et al., 2010; Kang et al., 2014), guinea pigs (Huang et al., 2014; Tan et al., 2016), dogs (Qiu et al., 2013), and mice (Lu et al., 2015). The observed reactions were dose-dependent (Qiu et al., 2013; Kang et al., 2014; Lu et al., 2015), and were also influenced by the injection speed (Lu et al., 2015), solvent type, and storage time of the preparation (Yi et al., 2015a). It remains controversial if SHLI-specific IgE evoked these reactions and the potential mechanisms related are still unclear. The present study aimed to explore the mechanism involved in SHLI-induced hypersensitivity reactions using mouse and cell models and elucidated the signaling pathway participated in these reactions. Based on the results of sensitization test, we proved that SHLI could induce IgE-independent but dose-related vascular hyperpermeability which was identical in SHLI-immunized and unimmunized mice. In addition, SHLI-specific IgE was not elicited by sensitization. Thus, our results validated the fact that SHLI evoked hypersensitivity reactions, which were pseudo-allergic, rather than IgE-induced allergic reactions.

The occurrence of SHLI hypersensitivity reactions were related to the level and rate of dose in clinical practice (Li, 2008; Yang et al., 2014). According to the information available in the instruction, the recommended clinical dose of SHLI for human is 60 mg/kg. Therefore, the highest dose of 600 mg/kg of SHLI used for mice in the present *in vivo* study was approximately the human equivalent clinical dose based on the equation for the conversion of animal doses (FDA, 2005). However, this corresponding dose concentration was five times higher than the maximum clinical value. Correspondingly, the dose concentrations for middle and low dose levels (300 and 150 mg/kg) were approximate 2.5 and 1.25 times as high as the clinical value, respectively. The dose rate designed for all animals was approximate 0.2 mL/min, which complied with the principles of animal welfare (Diehl et al., 2001). In the present research, treatment with high concentrations of SHLI resulted in profound EB leakage, and the extent of EB extravasation closely correlated with the dose concentration. Hence, we conclude that excessive dose concentration of SHLI could enhance vascular permeability, and trigger pseudo-allergic reactions. Therefore, establishment of safety profiles, development of reasonable medication regimens and control in administering methods can effectively alleviate SHLI-mediated hypersensitivity reactions.

Within the internal surface of blood vessels, adjacent endothelial cells are connected by junctional complexes and constitute a guardian monolayer. This layer plays a crucial role in maintaining the integrity and permeability of vascular walls. In paracellular pathway, cell junctions act as gatekeepers to control the passage of blood constituents and circulating cells between vasculature and the underlying tissues. The junctional complexes link to intracellular actin cytoskeleton and signal proteins (Hartsock and Nelson, 2008; Dejana et al., 2009), thereby stabilizing the extracellular adhesive contact between cells (Aghajanian et al., 2008; Nyqvist et al., 2008). Stimulation with inflammatory agents reorganizes the cytoskeletons and dissociates the endothelial junctions resulting in the breakdown of the endothelial barrier function and causing vascular leakage (Giannotta et al., 2013; Reglero-Real et al., 2016). In the present study, endothelial cells were incubated with SHLI and concentration-dependent stress fiber formation and distribution were observed. Our results indicated the ability of SHLI to induce separation of cell–cell contacts, leading to dysfunction of endothelial monolayer and facilitating transendothelial movement of fluid and plasma proteins, thereby inducing vascular leakage and inflammation in animals.

The RhoA/ROCK signaling pathway plays a pivotal role in regulating endothelial permeability. Activation of RhoA enhances the activity of ROCK and results in phosphorylation of MLC, which in turn widens the gap between neighboring endothelial cells through reassembly of actin cytoskeleton (Etienne-Manneville and Hall, 2002; Wallez and Huber, 2008; Zhao et al., 2015). In the present study, we prove the involvement of the RhoA/ROCK signaling pathway in SHLI-induced increased vascular permeability. The expression of GTP-RhoA, p-MYPT1, and p-MLC2 were significantly elevated in SHLI-incubated HUVECs, and were associated with reorganization of stress fibers. In the *in vivo* experiment, SHLI directly activated the RhoA/ROCK signaling pathway and led to inflammation and edema in the ears, lungs, and intestines of mice, which partly explain the reason of SHLI-induced ADRs seen in the skin, respiratory system, and gastrointestinal tract. In addition, a novel result of this study is the finding that fasudil could significantly protect against SHLI-induced vascular leakage. This might provide a potential therapeutic strategy for the treatment of SHLI-induced pseudo-allergic reactions.

In the form of a Chinese medicinal preparation, SHLI is a complex mixture composed of the extracts of *Flos lonicerae japonicae, Radix scutellariae*, and *Fructus forsythia*. Currently, more than 125 constituents have been identified from SHLI (Sun et al., 2015), of which chlorogenic acid, baicalin, and phillyrin are recorded as indicative components in Chinese Pharmacopeia. Several studies were initiated to find the chemical ingredients responsible for the hypersensitivity reactions induced by SHLI. Most clinicians considered chlorogenic acid as the chief culprit as its antigenic properties were identified since 1960s (Freedman et al., 1961; Freedman, 1964). In addition, most of the adverse reactions related to Chinese medicinal injections seemed to be because of chlorogenic acid containing preparations (Lin et al., 2012, 2013). Previous studies demonstrated that chlorogenic acid and its conversion product-cryptochlorogenic acid could directly evoke mast cell degranulation and β-hexosaminidase release, triggering pseudo-allergic reactions (Huang et al., 2010; Li et al., 2013; Wang et al., 2015). Similar outcomes were detected in the research related to baicalin (Han et al., 2014). Besides, forsythiaside A and arctiin, which are separated from forsythia are reported to cause pseudo-allergic reactions in mice (Yi et al., 2015b). Though diverse views related to the actual cause of hypersensitivity reactions exist (Yi et al., 2011; Feng et al., 2014), it is reasonable to speculate that chlorogenic acid, cryptochlorogenic acid, baicalin, forsythiaside A, and arctiin in SHLI may be responsible for inducing the pseudo-allergic reactions. Adopting stringent standards in quality control and strictly limiting component proportion during production are crucial in preventing ADRs caused by SHLI. Further studies are required to screen and identify the ingredients associated with SHLI-induced pseudo-allergic reactions.

## Author Contributions

The *in vitro* experiment and western blot assay were conducted by JH and HC. The *in vivo* experiment was performed by JH, YZ, YSZ, CL, YY, CP, and JL. The data were analyzed by LW, SL, and ND. The histopathological slides were examined by JT and YFY. JH and AL designed the entire study and wrote the manuscript.

## Conflict of Interest Statement

The authors declare that the research was conducted in the absence of any commercial or financial relationships that could be construed as a potential conflict of interest.
